# Perceval valve intermediate outcomes: a systematic review and meta-analysis at 5-year follow-up

**DOI:** 10.1186/s13019-023-02273-7

**Published:** 2023-04-11

**Authors:** Jarrod Jolliffe, Simon Moten, Amit Tripathy, Peter Skillington, James Tatoulis, Claudio Muneretto, Lorenzo Di Bacco, Hericka Bruna Figueiredo Galvao, John Goldblatt

**Affiliations:** 1 Cardiothoracic Department, Royal Melbourne Hospital, 300 Grattan Street Parkville, Melbourne, VIC Australia; 2 School of Cardiac Surgery, University of Brescia, Brescia, Italy; 3 Centre for Cardiovascular Biology and Disease Research, Latrobe University, Melbourne, VIC Australia

**Keywords:** Sutureless aortic valve replacement, Minimally invasive valve replacement, Rapid deployment aortic valve replacement

## Abstract

**Objectives:**

New technologies for the treatment of Aortic Stenosis are evolving to minimize risk and treat an increasingly comorbid population. The Sutureless Perceval Valve is one such alternative. Whilst short-term data is promising, limited mid-term outcomes exist, until now. This is the first systematic review and meta-analysis to evaluate mid-term outcomes in the Perceval Valve in isolation.

**Methods:**

A systematic literature review of 5 databases was performed. Articles included evaluated echocardiographic and mortality outcomes beyond 5 years in patients who had undergone Perceval Valve AVR. Two reviewers extracted and reviewed the articles. Weighted estimates were performed for all post-operative and mid-term data. Aggregated Kaplan Meier curves were reconstructed from digitised images to evaluate long-term survival.

**Results:**

Seven observational studies were identified, with a total number of 3196 patients analysed. 30-day mortality was 2.5%. Aggregated survival at 1, 2, 3, 4 and 5 years was 93.4%, 89.4%, 84.9%, 82% and 79.5% respectively. Permanent pacemaker implantation (7.9%), severe paravalvular leak (1.6%), structural valve deterioration (1.5%), stroke (4.4%), endocarditis (1.6%) and valve explant (2.3%) were acceptable at up to mid-term follow up. Haemodynamics were also acceptable at up mid-term with mean-valve gradient (range 9–13.6 mmHg), peak-valve gradient (17.8–22.3 mmHg) and effective orifice area (1.5–1.8 cm^2^) across all valve sizes. Cardiopulmonary bypass (78 min) and Aortic cross clamp times (52 min) were also favourable.

**Conclusion:**

To our knowledge, this represents the first meta-analysis to date evaluating mid-term outcomes in the Perceval Valve in isolation and demonstrates good 5-year mortality, haemodynamic and morbidity outcomes.

**Key question:**

What are the mid-term outcomes at up to 5 years follow up in Perceval Valve Aortic Valve Replacement?

**Key findings:**

Perceval Valve AVR achieves 80% freedom from mortality at 5 years with low valve gradients and minimal morbidity**.**

**Key outcomes:**

Perceval Valve Aortic Valve Replacement has acceptable mid-term mortality, durability and haemodynamic outcomes.

**Supplementary Information:**

The online version contains supplementary material available at 10.1186/s13019-023-02273-7.

## Background

Aortic Stenosis remains the most common valve pathology requiring intervention in developed countries, with significant morbidity and mortality if left untreated [1, 2]. Aortic Valve Replacement (AVR) remains the current treatment of choice, however an ageing population and increasing incidence of disease combined with increasing morbidity and potential surgical risk, has prompted the need for interventions which minimise surgical risk [3, 4]. The response to this has been the introduction of Trans-catheter Aortic Valve Replacement (TAVR) and Sutureless/Rapid-Deployment Aortic Valve Replacement (SURD-AVR) [5, 6]. Promisingly, SURD-AVR has demonstrated shortened cardiopulmonary bypass (CPB) times and aortic cross clamp times (ACC) both for isolated AVR and concomitant procedures [7, 8]. Post-operative outcomes remain comparable with those of standard surgical AVR (SAVR) with respect to mortality, complications and valve haemodynamics [7, 9, 10] Mid-term data had been lacking until Williams et al. conducted the first meta-analysis of mid-term outcomes in patients who had received SURD-AVR either with the Sutureless Perceval Valve, (Corcym SRL, Saluggia, Italy, previously LivaNova) or rapid deployment Intuity Valve (Edwards Life Sciences, California, USA) [11]. The analysis of four observational studies demonstrated satisfactory five-year survival for SURD-AVR, comparative to current survival seen in SAVR [11, 12]. Additionally, haemodynamic outcomes, whilst unable to be meta-analysed, were promising at five years and were once again comparable with other reported haemodynamic data for SAVR [11, 13, 14]. However, due to the limited published data available at that time, two studies only evaluating each valve were available for their analysis with limited numbers at follow-up, especially in papers analysing the Perceval Valve [15, 16]. With the publication of more mid-term data in this field, this systematic review and meta-analysis of published data aims to be the first to evaluate the mid-term outcomes and valve haemodynamics of the Perceval Valve with an increased number of patients at mid-term follow-up.

## Methods

### Literature search

A systematic review and Meta-Analysis of Perceval Valve implantation was undertaken in accordance with PRISMA guidelines. Five data bases were analysed—PubMED, SCOPUS, Cochrane Database, EMBASE and Ovid MEDLINE. Dates searched were from data-base inception to June 2022. Search terms utilised keywords in combination and MeSH headings. Headings utilised were rapid deployment AND Aortic Valve bioprosthesis or AVR, Sutureless AND Aortic Valve bioprosthesis OR AVR. Additionally, references from retrieved articles were assessed individually and included if inclusion criteria were met.

### Inclusion and exclusion criteria

Studies were included if patients had undergone AVR with a Perceval Valve either in isolation or with a concomitant procedure. Studies could be randomised control trials or observational studies. They had to have reported outcomes up to five years with full survival data required. Studies were excluded if they included valves other than the Perceval Valve or if there was insufficient time to event survival data (defined as up to five years). Grey literature was included as were studies not in English. Case reports, expert opinion, narrative reviews, abstracts and presentations were excluded.

### Data extraction

Data was extracted directly from texts, tables and Additional file 1. Where data was incomplete, authors of publications were emailed directly. All retrieved articles were reviewed by two reviewers (JJ and AT). Disagreements between reviewers were discussed with a third reviewer (JG) and if required, discussions were conducted amongst all investigators.

### Outcomes and statistical analysis

The primary outcome of interest was survival and haemodynamic performance at five years. Secondary outcomes included: early and late post operative mortality and morbidity, early and late re-intervention and valve explantation/TAVR intra-operative failure and CPB and ACC times. Using a random effects model, dichotomous and continuous outcomes were pooled to provide either weighted averages or proportion with a reported 95% Confidence Interval (CI). Heterogeneity was calculated with an I^2^ statistic with minimal heterogeneity being between 0–49%, moderate between 50 and 74% and high being > 75%. Funnel plots would be utilised to assess publication bias. The method utilised by Williams et al. and devised by Guyot et al. to aggregate overall survival was instituted [11, 17]. This generated individual patient data from digitised Kaplan–Meier curves and created an aggregate survival curve. DigitiseIT (DigitalRiver GmbH, Cologne, Germany) was used to extrapolate the digitised curves. Comprehensive Meta-Analysis Software V3.3 (Biostat Incorporated, NJ, USA) was utilised in the analysis of dichotomous and continuous outcomes. Quality of studies was reviewed with the Cochrane GRADE system, with bias assessed through the Risk of Bias in Non-Randomised Studies of Interventions tool (ROBINS-I) [18, 19].

## Results

### Quality of evidence

**Fig. 1:**
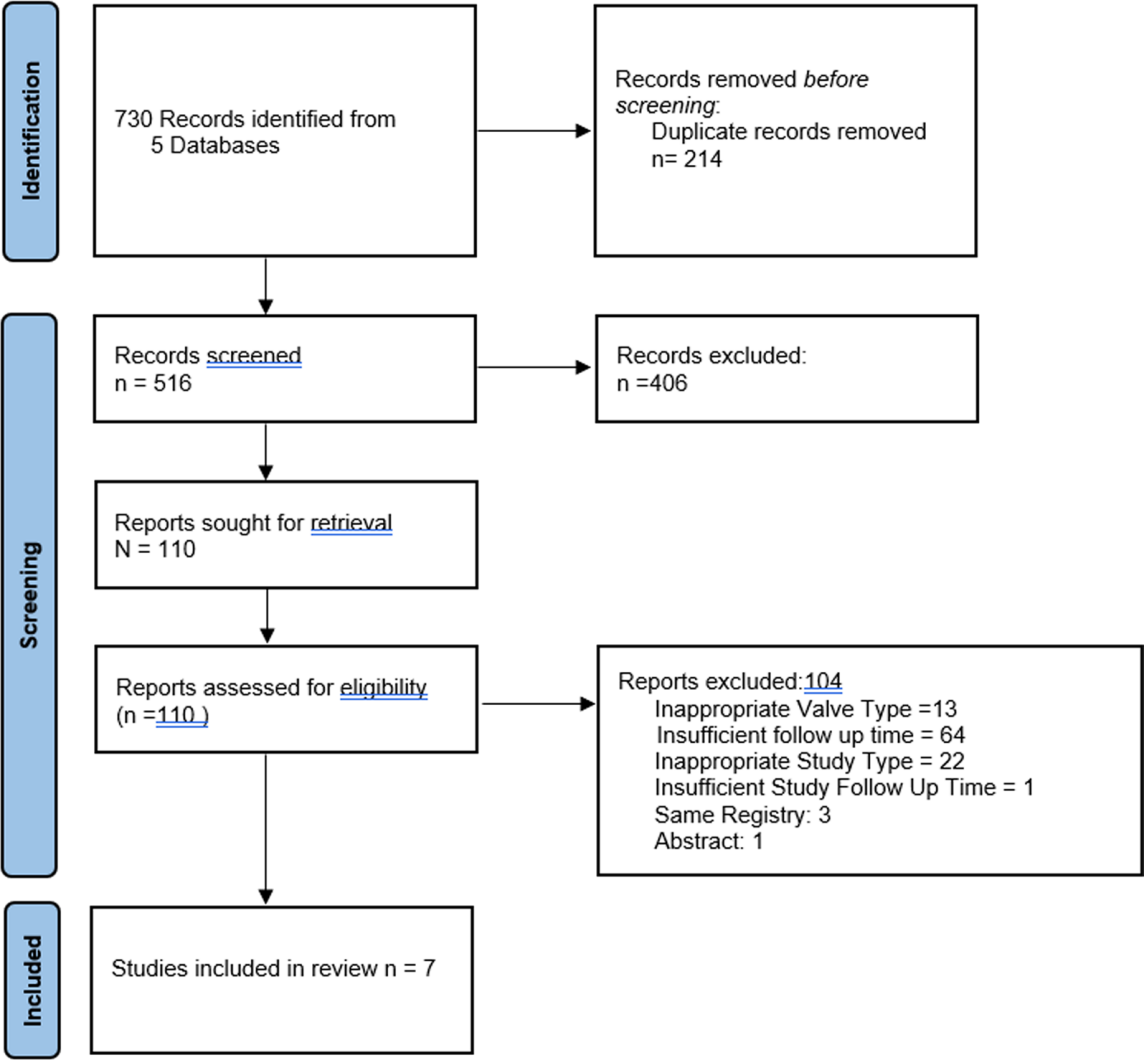
PRISMA flow sheet outlining publication search strategy

The study selection process is outlined in Fig. 1. Initially, 730 articles were identified and after removal of duplicates and exclusion of inappropriate articles, 110 were identified for full review. In total, 104 articles were excluded. Three studies were excluded due to overlap with registry data from the final included papers [20–22]. One study was excluded as it was a presentation with only the abstract available [23]. Seven studies were included in the final analysis with a combined patient number of 3196 patients [9, 16, 21, 24–27].

All seven studies were observational, with three prospective cohort studies [16, 21, 25] and four prospective cohort studies, with mean follow up time between 1.8 and 7 years [9, 24, 26, 27]. Refer to Table 1 for details. Three of the studies included complete echocardiographic data at five years [21, 24, 28] whilst one did not contain peak valve gradient (PVG) data [16]. Two of the included studies did not report complete echocardiographic data but did include latest follow up echocardiographic data with a mean follow-up time of 3 years and maximum of 11.2 years [27] and median of 2.6 years with a maximum of 13.5 years [26]. One study failed to include echocardiographic data [9]. In three of the four studies, echocardiographic data was collected independently and analysed [21, 24, 25] whilst in one, it was institutional data [16]. All studies reported five-year mortality estimates with at-risk data provided. The three prospective studies were, non-randomised single arm studies [16, 21, 25]. One of the retrospective studies compared TAVR with the Perceval Valve, one compared SAVR with the Perceval Valve whilst the remaining two evaluated the Perceval Valve in isolation [9, 24, 26, 27]. Four of the studies were multi-centre, with three of these studies evaluating data across multiple centres in Europe [21, 24, 25] and one multi-national trial [16]. The remaining three were single-centre [9, 26, 27]. All studies, except Glauber et al., provided explicit inclusion and exclusion criteria [9, 16, 21, 24–27]. Loss to follow-up was not clearly reported in three of the four studies [16, 21, 26]. Fischlein et al. suffered significant attrition losing 293 of the 628 patients who underwent valve replacement, whilst three of the studies had near complete follow up data [9, 24, 25, 27]. Only one study included final follow-up numbers for echocardiographic data [25]. Authors from 6 of the 7 studies disclosed a conflict of interest with LivaNova, now owned by Gyrus Capital and run by CORCYM, either financially as consultants or as recipients of research grants [9, 16, 21, 24–27]. Significant heterogeneity was encountered in key outcomes such as mid-term mortality and PVL with moderate heterogeneity encountered with SVD, explantation/reintervention and pacemaker implantation. Funnel plots were not utilised as there were less than 10 studies included in this meta-analysis.

**Table 1:** Summary of quality of evidence. ROBINS-I (risk of bias in non-randomised studies tool), GRADE (grading of recommendations, assessment, development and evaluation)

Papers	Study design	Trials/registries	Patient number	Median follow up (years)	Loss to follow up	Confounders factors identified	Risk of bias (ROBINS-I)	GRADE sore
Meuris et al. (2015)	Prospective Cohort	Perceval Pilot Trial (5 years)	30	5	Unclear	No	Severe	+ + + Moderate
FIschlein et al. (2021)	Prospective Cohort	CAVALIER	658	3.8	46.7%	No	Severe	+ + + Moderate
Glauber et al. (2019)	Prospective Cohort	SURE-AVR Registry	480	2.4	Not clearly Stated	No	Severe	+ + + Moderate
Muneretto et al. (2022)	Retrospective Cohort	None: Institutional Data	481	5	No Loss to follow up	Yes	Moderate	+ + + Moderate
White et al. (2022)	Retrospective Cohort	None: Institutional Data	295	2.4	No Loss to follow up	Yes	Severe	+ + Low
Szecel et al. (2021)	Retrospective Cohort	None: Institutional	468	3.1	1% loss to follow up	No	Severe	+ + + Moderate
Lamberigts et al. (2022)	Retrospective Cohort	None: Institutional	784	7	Unclear	No	Severe	+ + + Moderate

### Demographics

Baseline demographics are reported in Table 2. The weighted average age was 78 with the population being 44% male and 56% female. Only study included weight, height, body-surface area (BSA) and only two reported BMI and so neither was pooled. The pooled population was relatively free of comorbidities. Chronic lung disease and CKD were the most reported comorbidities (16% and 9% respectively), followed by diabetes and peripheral arterial disease. Euroscore-1 was reported in four studies, with a weighted average of 11 (95% CI 8.8–13.3) whilst Euroscore-2 was reported in two studies both of which had low risk scores. NYHA III/IV symptoms were seen in 53.9% (44.6–63.1) of the population. Due to a lack of reporting in 3 out of the four studies, surgical characteristics were unable to be pooled. In those that reported it, 93–100% of valves were tricuspid and between 2 and 10% were bicuspid [16, 21, 24, 28]. Four studies reported the indication for surgery, with aortic stenosis or mixed aortic stenosis/insufficiency the primary indication in 98–100% of cases[16]. Whilst data was not provided, Scezel et al. also noted that < 1% of patients underwent valve replacement for pure aortic insufficiency [27]. Pre-operative surgical characteristics are summarised in Table 3. Pre-operative haemodynamics were unable to be pooled as only three studies reported on this data [16, 24, 28]. Where reported, MVG was between 45 and 49.3 mmHg, PVG was between 73.5 and 78.2 mmHg and EOA was 0.7. Mean LVEF in three studies was between 58 and 63% [21, 24, 28], whilst 68–80% of patients between three studies having an LVEF of > 50% [9, 26, 27]. Pre-operative haemodynamic data is summarised in Table 4.

**Table 2:** Demographic data: NYHA (New York Heart Association), STS (Society of Thoracic Surgeons), HTN (Hypertension), TIA (Transient Ischaemic Attack), CKD (Chronic Kidney Disease), PAD (Peripheral Arterial Disease) CAD (Coronary Artery Disease)

Study	Patient No	Age	Gender (male)	NYHA I	NYHA II	NYHA > III	Euro-score	Euroscore II	STS-score	HTN	Cholesterol	Smoker	Diabetes	Stroke/TIA	Chronic lung disease	CKD	PAD	CAD
Meuris et al. 2016	30	80.4 + − 3.8	22 (7.3%)	N/A	N/A	N/A	13.18 + − 7.3	N/A	N/A	N/A	N/A	N/A	N/A	N/A	N/A	N/A	N/A	N/A
FIschlein et al. 2021	658	78.3 +− 5.6	234 (35.6%)	22 (3.3%)	198 (30.1%)	418 (53.5%)	10.2 + − 7.8	N/A	72 + − 7.4	551 (83.74%)	N/A	31	191 (29.02%)	75 (11.4%)	103 (15.7%)	97 (14.7%)	112 (17.02%)	N/A
Glauber et al. 2020	480	76 +− 6.8	171 (35.6%)	19 (3.9%)	284 (59.2%)	171 (35.6%)	7.9 +− 12.3	N/A	N/A	N/A	291	N/A	139 (28.9%)	27 (5.6%)	80 (16.7%)	40 (8.3%)	N/A	83 (17.2%)
Muneretto et al. 2022	481	79 +− 5	174 (36.2%)	N/A	N/A	285 (59.9%)	13.6 +− 18.4	N/A	5.7 + − 6.4	394 (81.93%)	N/A	N/A	154 (32.01%)	31 (6.4%)	89 (18.5%)	59 (12.8%)	81 (16.8%)	143 (29.7%)
White et al. (2022)	295	72.4 +− 9.9	188 (63.7%)	N/A	N/A	N/A	N/A	N/A	N/A	180 (61%)	166 (56.3%)	58 (19.7%)	75 (25.4%)	14 (4.7%)	40 (13.6%)	12 (4.1%)	N/A	61 (20.7%)
Szecel et al. (2021)	468	79 + − 5	206 (44%)	26 (5.6%)	164 (35%)	278 (59.4%)	N/A	5.1 _ 5.5 (0.8–67)	N/A	N/A	N/A	N/A	N/A	N/A	75 (16%)	28 (6%)	122 (26%)	99 (21.2%)
Lamberigts et al. (2022)	784	78.5 +− 5.8	279 (48.3%)	45 (5.7%)	333 (42.5%)	406 (51.8%)	N/A	4.2 (2.6–7.2)	N/A	N/A	N/A	N/A	N/A	N/A	119 (15.2%)	27 (3.4%)	196 (25%)	62 (7.9%)
Weighted average/total	3196	77.7 (76.5–78.8)	44.4% (36.9–51.8)	4.2% (3.1–5.6)	37.5% (26–49.7)	53.9 (44.6–63.1)	11.04 (8.8–13.3)	N/A	N/A	N/A	N/A	N/A	29.2% (26.9–31.5)	7.7% (5.4–10.4)	16% (14.52–17.3)	8.6% (5.2–12.8)	20.1% (16–24.4)	18.8% (11.3–27.7)
I2	N/A	63.3%	94.2%	67.5%	98%	96.13%	29%	N/A	N/A	N/A	N/A	N/A	23%	83.2%	16.92%	94.5%	88.9%	96.5%

**Table 3:** Pre-operative surgical characteristics: TAV (tricuspid aortic valve), BAV (bicuspid aortic valve), FS (full-sternotomy), Mini-Inv (minimally invasive), AS (aortic stenosis), AR (aortic regurgitation)

Study	TAV	BAV	Prosthesis	FS	Mini-Inv	Minithoracotomy	Ministernotomy	Re-Sternotomy	Prior CABG	Prior Valve	AS	AS/AR	**AR**
Meuris et al. (2015)	30 (100%)	0	0	30 (100%)	0	0	0	3 (10%)	N/A	N/A	23 (76.6%)	7 (23.3%)	0
FIschlein et al. 2021	658 (96.96%)	12 (1.8%)	9 (1.4%)	439 (66.7%)	219	216	3	34 (5.2%)	13 (1.98%)	11 (1.7%)	359 (54.6%)	226 (34.34%)	2 (0.3%)
Glauber et al. 2020	433 (90.2%)	47 (9.8%)	8 (1.7%)	0	480	266	214	20 (4.2%)	5 (1.04%)	15 (3.1%)	430 (89.6%)	107 (22.3%)	11 (2.3%)
Muneretto et al. 2022	450 (93.6%)	31 (6.5%)	N/A	256 (53.2%)	225	94	131	35 (7.3%)	18 (3.7%)	N/A	481 (100%)	0	0
White et al. (2022)	N/A	N/A	N/A	N/A	N/A	N/A	N/A	N/A	10 (3.4%)	N/A	N/A	N/A	N/A
Szecel et al. (2021)	N/A	11 (2.4%)	N/A	328 (70%)	140 (30%)	14	126	N/A	N/A	N/A	N/A	N/A	8 (1.7%)
Lamberigts et al. (2022)	N/A	N/A	N/A	541 (69%)	243 (31%)	16	227	N/A	N/A	N/A	N/A	N/A	N/A
Weighted average/total	N/A	N/A	N/A	58.43% (27.5–86.1)	41.6% (14–72.6)	14.51% (2.4–34.5)	17.5% (5–35.7)	N/A	N/A	NA	N/A	N/A	N/A
I2	N/A	N/A	N/A	99%	99%	99.4%	99.2%	N/A	N/A	N/A	N/A	N/A	N/A

**Table 4:** Pre-operative haemodynamic data: MVG (mean valve gradient), peak valve gradient (PVG), EOA (effective orifice area), LVEF (left ventricular ejection fraction)

Study	MVG (mmHg)	PVG (mmHg)	EOAcm^2^	LVEF%
Meuris et al	N/A	N/A	N/A	63
FIschlein et al. (2021)	45 +− 15.9	73.5 + − 24.9	0.7 +− 0.2	56.6
Glauber et al. (2020)	49.3 + − 14.6	N/A	0.7 + − 0.2	N/A
Muneretto et al. (2022)	47.9 +− 16.6	78.2 + − 25.2	0.7 + − 0.2	57.7
White et al. (2022)	N/A	N/A	N/A	N/A
Szecel et al. (2021)	N/A	N/A	N/A	N/A
Lamberigts et al. (2022)	N/A	N/A	N/A	N/A
Weighted average (95% CI)	N/A	N/A	N/A	N/A

### Intra-operative outcomes

Intra-operative findings are summarised in Table 5. Of the remaining 2318 patients, 71.62% (47.1–90.7) underwent isolated AVR and 27.6% (8.9–51.8) underwent a concomitant procedure. With respect to surgical access, 58.43% (27.5–86.1) of patients underwent full sternotomy 41.6% (14–72.6) underwent a minimally invasive approach with 17.5% undergoing ministernotomy and 14.5% undergoing right anterolateral thoracotomy. Pooled ACC times for AVR were 52 (43.31–60.6) whilst CPB times were 78.1 (67.8–88.4). Pooled ACC times for isolated procedures were 35.71 (33.64–37.8) and for CBP were 57.7 (52.6–62.8). Concomitant ACC and CPB times were unable to be pooled due to insufficient data, however concomitant ACC times were reportedly between 45 and 79 min whilst CPB times were between 73.4 and 118 min [21, 27, 28]. The most implanted valves sizes were medium and large with weighted averages of 49% (37.7–60.3) and 33.6% (26.8–40.73) respectively. Small and extra-large valves were seldom implanted in 9.2% (5.6–13.7) and 11% (4.6–19.7) respectively.

**Table 5:** Intra-operative data: ACC (aortic cross-clamp time), CPB (cardiopulmonary bypass time), S (small), M (medium), L (large), XL (extra-large)

Study	Isolated AVR	Concomitant AVR	Avg ACC (Min)	Iso ACC (Min)	Concomitant ACC (Min)	Avg CPB (Min)	Iso CPB (Min)	Concomitant CPB (Min)	S	M	L	XL	Intraoperative failure
Meuris et al. (2015)	16 (53%)	14 (46%)	N/A	29.3 + −8.0	45.4 + −15.4	N/A	46.4 + −6.7	73.4 + −21.8	0	30 (100%)	0	0	0
FIschlein et al. 2021	418 (63.6%)	210 (31.9%)	40.7 + − 18.1	35.5 +− 12.4	52.3 +− 22.9	64.8 + −25.2	58.7 +− 20.2	78.7 +− 29.4	84 (13.4%)	290 (46.2%)	255 (40.6%)	29 (4.6%)	30 (3.1%)
Glauber et al. 2020	457 (95.21%)	23 (4.8%)	51 +− 17	N/A	N/A	81 +− 36.7	N/A	N/A	72 (16%)	151 (31.5%)	203 (42.3%)	54 (11.3%)	N/A
Muneretto et al. 2022	481 (100%)	0	35 + −16	35 +− 16	N/A	56 +− 25	56 +− 25	N/A	N/A	N/A	N/A	N/A	N/A
White et al. (2022)	201 (68.1%)	90 (31%)	73.8 +− 37.5	N/A	N/A	108.3 +− 56.4	N/A	N/A	N/A	N/A	N/A	N/A	N/A
Szecel et al. (2021)	201 (45%)	267 (57%)	61 +− 30	39 + −13	79 +− 32	94 +− 40	66 + −22	118 + −40	29 (6%)	160 (34%)	175 (37%)	104 (22%)	N/A
Lamberigts et al. (2022)	349 (45%)	435 (55%)	51 +− 34.8	38 (32–45)	N/A	81 +− 61–119	61 +− 51–72.8	N/A	63 (8%)	267 (34.1%)	291 (37.1%)	163 (20.8%)	21 (2.7%)
Weighted average	71.62% (47.1–90.7)	27.6% (8.9–51.8)	52 (43.31–60.6)	35.71 (33.64–37.8)	N/A	78.1 (67.8–88.4)	57.7 (52.6–62.8)	N/A	9.2% (5.6–13.7)	49% (37.7–60.3)	33.6% (26.8–40.73)	11% (4.6–19.7)	N/A
I2	99.51%	99.5%	40%	55.35%	N/A	43.4%	35%	N/A	90.2%	96.5%	91.4%	96.9%	N/A

### Mid-term mortality and haemodynamics

**Fig. 2:**
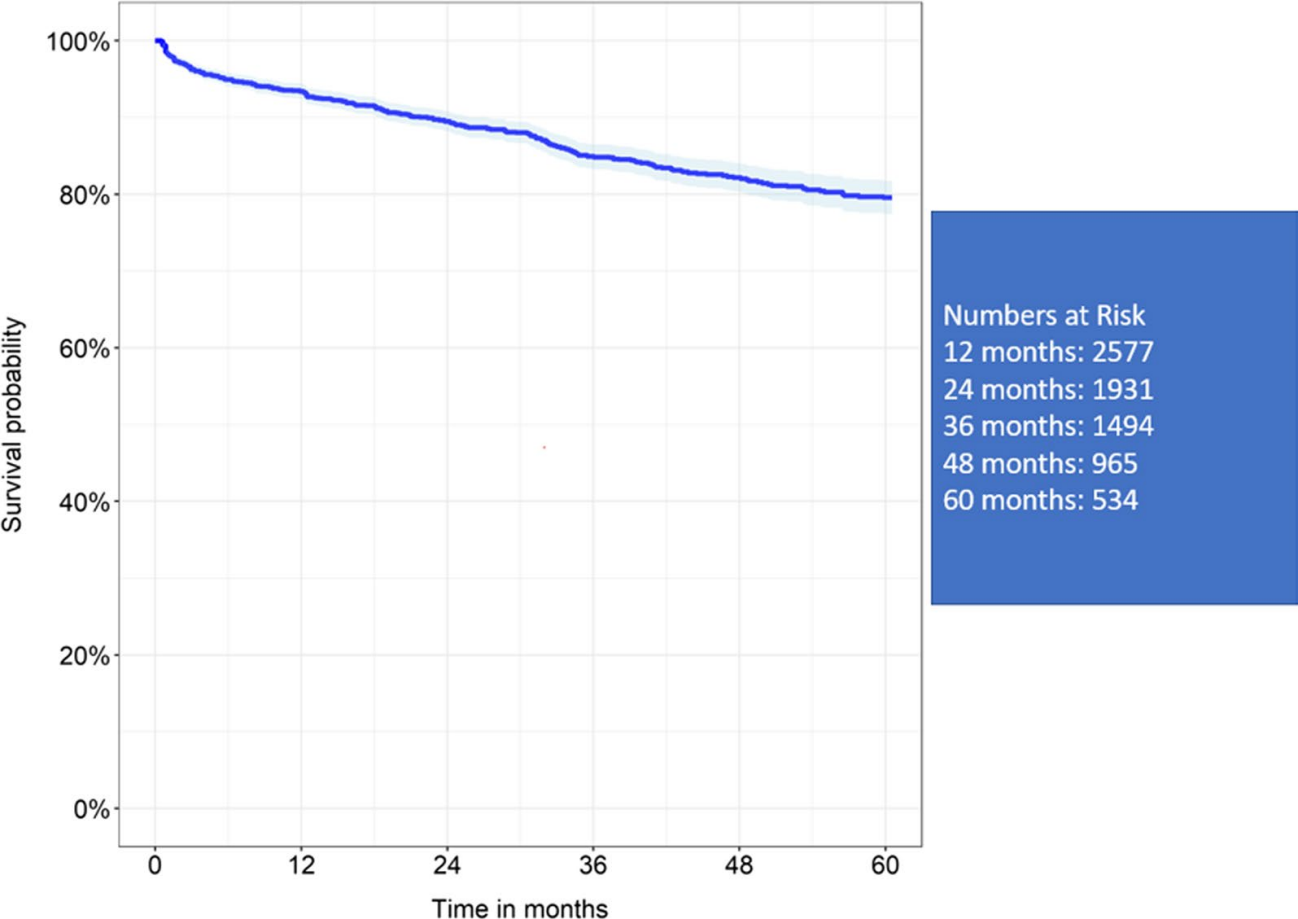
Reconstructed Kaplan Meier curve of 5-year mortality data

Survival at 1, 2, 3, 4 and 5 years was 93.4%, 89.4%, 84.9%, 82% and 79.5% (refer to Fig. 2).

Weighted pooled estimates for overall mortality with a mean follow up of 4.1 years was 11.2% (4.1 to 21.3). Echocardiographic data was unable to be pooled as studies did not provide patient numbers for each time point and only four studies included full echocardiographic data as seen in Table 6 [16, 21, 24, 28]. Discharge MVG ranged from 6.9 mmHg to 13.9 mmHg whilst each time point between one and five years remained at a range between 7.7–13.6 mmHg. Post-discharge PVG ranged between 14.5 mmHg and 23.2 mmHg whilst the one–five-year ranges were between 17.1 mmHg and 22.3 mmHg. Scezel et al. with 3 years mean follow up had MVG and PVG of 13 mmHg and 23 mmHg respectively [27]. Lamberigts et al. with 2.6 years median follow-up had median MVG and PVG of 11 and 20 mmHg [26]. EOA remained between 1.5–1.7cm^2^ at discharge, whilst between one and five years ranged between 1.5 and 1.8 cm^2^.

**Table 6:** Summary of mid-term echocardiographic data: MVG (mean valve gradient), Peak valve gradient (PVG), EOA (effective orifice area), LVEF (left ventricular ejection fraction)

	Post-operative			1 year			2-year		
Study	MVG (mmHg)	PVG (mmHg)	EOAcm^2^	MVG (mmHg)	PVG (mmHg)	EOAcm^2^	MVG (mmHg)	PVG (mmHg)	EOAcm^2^
Meuris et al. (2015)	N/A	N/A	N/A	9.9 + −4.6	20.9 + −9.2	1.55 + −0.35	8 + −4.1	16.6 + −7.2	1.51 + −0.26
FIschlein et al. (2021)	10.3 +− 4.5	19.4 +− 8	1.5 +− 0.4	9.1 +− 5.0	17.1 + −8.7	1.5 +− 0.4	9.3 +− 4.8	17.1 +− 8.4	1.5 +− 0.4
Glauber et al. (2020)	13.9 +− 4.7	19.4 +− 8	1.7 +− 0.4	11.6 +− 5.1	N/A	1.7 + −0.5	11.3 +− 5.4	N/A	1.6 + −0.4
Muneretto et al. (2022)	11.1 + −5.7	23.2 +− 9.3	1.5 +− 0.4	10.9 +− 5.9	21.26 + −9.5	1.54 + −0.31	11.2 +− 6.1	21.5 +− 9.2	1.53 +− 0.31
White et al. (2022)	6.9 +− 4.1	14.5 +− 7.7	N/A	N/A	N/A	N/A	N/A	N/A	N/A
Szecel et al. (2021)	15.3 +− 5.8	27.9 +− 10.1	N/A	N/A	N/A	N/A	N/A	N/A	N/A
Lamberigts et al. (2022)	14 (11–18)	25 (20–32)	1.6 (1.3–1.9)	N/A	N/A	N/A	N/A	N/A	N/A

	3-year			4- year			5-year		
Study	MVG (mmHg)	PVG (mmHg)	EOAcm^2^	MVG (mmHg)	PVG (mmHg)	EOAcm^2^	MVG (mmHg)	PVG (mmHg)	EOAcm^**2**^
Meuris et al. (2015)	8.3 + −2.5	16.6 + −6.2	1.68 + −0.4	7.6 + −3.6	17.5 + −7.8	1.68 + −0.43	9.3 + −5.5	21.4 +− 11.5	1.69 +− 0.42
FIschlein et al. (2021)	9.3 +− 5.8	17.2 +− 9.8	1.5 +− 0.4	9.6 +− 5.7	18.5 +− 10.4	1.5 +− 0.3	9.0 +− 6.3	17.8 +− 11.3	1.5 +− 0.5
Glauber et al. (2020)	11.3 +− 5.4	N/A	1.4 + −0.4	12.6 +− 6.2	N/A	1.5 + −0.4	13.6 + −8.6	N/A	1.5 + −0.5
Muneretto et al. (2022)	11.6 +− 5.8	21.9 +− 9.1	1.50 +− 0.31	12 +− 5.4	22.1 +− 9.1	1.45 +− 0.32	12.2 +− 5.7	22.3 +− 9–0	1.42 + −0.31
White et al. (2022)	N/A	N/A	N/A	N/A	N/A	N/A	N/A	N/A	N/A
Szecel et al. (2021)	N/A	N/A	N/A	N/A	N/A	N/A	N/A	N/A	N/A
Lamberigts et al. (2022)	N/A	N/A	N/A	N/A	N/A	N/A	N/A	N/A	N/A

### Mid-term morbidity outcomes

Mid-term outcomes up to five years are summarised in Table 7. Weighted pooled estimates for follow-up, up to five years, demonstrated PVL rates 3.6% (95% CI 2.2–5.4%) whilst severe PVL was 1.6% (95% CI 0.7–2.7%), although the definition of this was inconsistently reported. SVD occurred in 1.5% (95% CI 0.7–2.6%) with less than 1% of valves replaced or reintervened upon because of SVD. Mid-term weighted estimates for stroke were 4.4% (95% CI 3.2–6%) whilst infective endocarditis was only 1.6% (95% CI 0.72–2.9%). Permanent Pacemaker Implantation (PPI) had a weighted pooled average of 7.9% (95% CI 5.6–10.5%). A weighted average of 2.3% (95% CI 1.3–3.4%) of valves were explanted, with endocarditis the most common reason for explantation.

**Table 7:** Mid-term mortality and morbidity data: IE (infective endocarditis), PPI (permanent pacemaker implantation), SVD (structural valve deterioration), PVL (paravalvular leak)

	Total	Weighted average	I2 (%)
Mortality	354/2382	11.2% (4.1–21.3)	97.7
SVD	39/2408	1.5% (.7–2.6)	68.1
PVL	97/2901	3.6% (2.2–5.4)	79.3
PVL severe	39/2403	1.6% (0.7–2.7)	68.2
Explant/valve in valve	70/2901	2.3% (1.3–3.4)	69.2
Explants due to PVL	19/2901	0.6% (01–1.5)	81.4
Explants/valve in valve due to SVD	17/2403	0.4% (0.05–1.2)	79.2
Explants due to Endocarditis	29/2403	1.1% (0.72–1.5)	0
PPI	171/2087	7.9% (5.6–10.5)	73.7
Stroke	118/2698	4.4% (3.2–6)	58.1
IE	50/2871	1.6% (0.72–2.9)	79

### Early post-operative outcomes

Early mortality of less than thirty days had a weighted average of 2.5% (05% CI 1.8–3.3%). Post-operative stroke occurred with a weighted estimate of 2.1% (95% CI 1.7–2.7%). Early PPI had a weighted average of 6.8% (95% CI 5.2–8.6%). Explantation occurred in less than 1% of cases, with paravalvular leak responsible for almost 90% of those cases. Notably, 56% of cases of severe PVL occurred within the first 30 days of operation with 90% of explantations/reinterventions due to PVL occurring within those first 30 days (Table 8). Reasons for paravalvular leak in the early period were provided in one study only with malposition the most cited reason [24].

**Table 8:** Short-term outcomes < 30 Days: IE (infective endocarditis), PPI (permanent pacemaker implantation), SVD (structural valve deterioration), PVL (paravalvular leak)

	Total	Weighted average	I2
Mortality	79/3166	2.5% (1.8–3.3)	47.6%
Stroke	65/3196	2.1% (1.7–2.7)	0
PVL	27/3196	0.9% (0.55–1.3)	26.4%
Severe PVL	22/3196	0.7% (0.3–1.2)	58.5%
Explants	19/2871	0.6% (0.097–1.5)	81%
Explants due to PVL	17/2901	0.5% (0.09–1.3)	79.3%
PPI	224/3196	6.8% (5.2–8.6)	69.5%

## Discussion

With increasingly comorbid patients, Aortic Valve disease and its management continue to evolve and with it, the need for procedures that minimise procedural risk. The invent of SURD-AVR addresses this through the minimisation of tissue manipulation, cross clamp and bypass times and its excellent haemodynamics even in small aortic annuli [7, 29, 30]. Whilst numerous studies in the literature report the intraoperative, immediate post-operative data and short-term data, few have addressed outcomes beyond five years. Whilst Williams et al. published the first meta-analysis evaluating intermediate outcomes in all SURD-AVR, this review, to our knowledge, is the first to evaluate intermediate outcomes specifically in the Perceval Valve and adds to a growing body of literature outlining the safety and effectiveness of these valves [11].

With respect to the primary outcome, five-year aggregate freedom from mortality in this study was 79% which is similar to that found in the current literature for SAVR, which has been reported repeatedly between 75% and 86% [31–34]. Whilst meta-analysis was unable to be conducted for haemodynamic data, performance across the four studies at five years was promising with MVG, PVG and EOAs ranging between 7.7–13.6 mmHg, 14.5–23.2 mmHg 1.5–1.8cm^2^respectively [16, 21, 24, 28]. This remains comparable with data evaluating Stented and Stentless Bioprosthetic Valves at five years with MVG’s between 8 and 18 mmHg, PVG between 15 and 30 mmHg and EOA of 1.4–1.6cm^2^ and [35–38]. The Perceval Valve differs from a Stented Valve due to its lack of sewing ring. This affords the valve a larger EOA. For this reason, proponents for the Perceval Valve note one of its significant advantages is in the patient with the small aortic annulus [39, 40]. Observational studies have demonstrated reduced incidence of short-term patient prosthesis mismatch and haemodynamics compared with Stented Valves in this cohort, whilst the comparable short-term outcomes with Stentless Valves were offset by the Perceval Valve’s significantly reduced intra-operative times [40, 41]. Unfortunately, the studies evaluated in this review did not focus on this patient population with 75% receiving medium, large and extra-large valve sizes. Consequently, mid-term haemodynamic and outcome data remain elusive in this group.

Mid-term performance was acceptable as severe PVL and SVD remained low with pooled averages of 1.6% and less than 1.5% respectively. SVD remained comparable with the current literature, with freedom from SVD at 5 years between 98–100% [42–45]. PVL rates remained low in our review with a pooled estimate of 3.6%. This is higher than the PVL leak rates reported in conventional bioprosthetic valves, which is between 0 and 1% but lower than those reported for TAVR, which is between 3 and 25% [45–50]. At five years, between 15 and 30% of TAVR patients will have mild to severe PVL, with PVL an independent risk factor for mid-term mortality [38, 47, 51]. Weighted PVL rates in the current study, were less than those found in the Williams et al. meta-analysis (9.2%), whilst severe PVL remained similar [11]. This difference is difficult to interpret but may be the result of the inclusion of the Intuity Valve in their analysis. Increased rates of PVL in the Intuity valve when compared to SAVR have been demonstrated in the CADENCE-MIS trial whilst a 2020 meta-analysis demonstrated a 3.3% re-intervention rate due to PVL in those who received an Intuity Valve[52, 53]. In the current analysis, re-intervention rates secondary to PVL were less than 1%. Whilst annular asymmetry and geometry have been proposed as potential mechanisms for the rates of PVL in Intuity Valve patients, such findings wouldn’t be unique to the Intuity Valve, especially given the Perceval’s lack of sutures and inability to reshape the aortic annulus, without modifying the technique of implantation [54, 55]. An advantage of the Perceval Valve is its collapsed configuration prior to deployment which allows direct visualisation and confirmation of position prior to deployment. Following deployment, this visualisation of and flexibility in the stent, allows for small adjustments to be made within the annulus to prevent or avoid PVL and if not correctly positioned, facilitates easy removal. Whilst the inclusion of the Intuity Valve in the Williams et al. analysis may explain the increased PVL rate, this didn’t seem to impact their rate of explant which was identical to that found in this analysis [11]. However, Flynn et al.’s 2020 meta-analysis comparing the Intuity Valve with the Perceval Valve, found a statistically significant increased incidence of overall post-operative PVL in the Perceval group, however no difference was seen in moderate or severe rates of PVL [56]. Currently, no comparative data evaluating mid to long-term outcomes in these valves exists, and to date, there are no randomised studies evaluating their short-term outcomes. Subsequently, direct comparative trials of anatomically and geometrically similar groups would be required to determine the true differences in observed leak rates.

Post-operative stroke and infective endocarditis at both early and mid-term follow up remained similar to those reported in the literature SAVR [7, 12, 57]. With respect to TAVR, stroke rates in the post-operative period are similar to those recorded in this analysis, with rates between 2–5% seen in the literature comparing SURD-AVR and TAVI [49, 58]. Whilst in the short-term stroke rate appears comparable to SURD-AVR, recorded rates at five years are between 10 and 15%, much higher than the 4% seen in this analysis [38, 59, 60]. Pacemaker implantation remains a consideration for Sutureless Valves with a weighted pooled estimate of 7.9%, which is similar to what has been reported in the literature for Perceval Valves, with rates between 4 and 10% [61–63]. Factors such as intra-annular placement, oversizing and sub-annular nitinol frame protrusion have been suggested as possible mechanisms for Atrioventricular (AV) nodal blockade and requirement for PPI [64–66]. Like others, our sizing and implantation technique has evolved over time, with guiding thread placement at or close to the annulus and avoidance of over-sizing now routinely utilised to mitigate the risk of AV blockade and PPI insertion [65, 67]. Furthermore, several studies have noted that pre-operative right bundle branch block (RBBB) and QRS prolongation are independent risk factors for PPI. Subsequently, we use careful consideration before these patients proceed to Perceval Valve implantation [68–70]. More recently one author (SM) has routinely used pre-operative computerised tomography (CT) Aortogram (TAVI protocol) measurements of aortic annular perimeter and area to guide intra-operative Perceval sizing, derived from Perceval sizing measurements of the inflow ring diameter “out of the jar”[71]. This has routinely simplified sizing and eliminated concerns of oversizing and pinwheeling of leaflets as well as reducing overall pacemaker rates. Use of CT measurements to predict AV sizing is growing in stature. The group in Massa have recognized that oversizing of the Perceval Valve by more than 30%, based on pre-operative aortic annular area led to an increased likelihood of increased trans-prosthetic gradients and stent infoldings [72]. Park et al., have suggested the use of pre-operative CT-based sizing for the Intuity Elite Valve. They found the CT based AV annulus dimensions and left ventricular outflow tract dimensions predicted AV blockade, PPI and PVL when using the Intuity Valve[73]. Based on our experience in Melbourne, pre-operative CT is used whenever possible prior to Perceval Valve implantation and has assisted in sizing, access and appropriate patient selection.

Extended ACC and CPB times have been associated with increased morbidity and mortality post-operatively [74, 75]. An advantage of the Perceval Valve is its rapidity of deployment and reduced tissue manipulation. This analysis echoes these findings with a weighted ACC and CPB time of 52 and 82 min respectively, in all cases, and 38 and 61 min in isolated AVR cases. It is reflective of the current literature with ACC times cited between 17 and 60 min and CPB times between 35 and 90 min [76]. However, with adequate myocardial protection likely suited to dealing with higher risk patients undergoing isolated AVR, the real benefit will be reflected in those undergoing concomitant procedures where prolonged ACC and CPB times may become unavoidable [77–79]. Three of the seven studies captured data on patients undergoing concomitant procedures [16, 25, 27]. Concomitant ACC times were similar to those found in the literature which are reportedly between 30–70 min and 44–88 min for ACC and CPB respectively [76]. Unfortunately, there remains a significant lack of data evaluating concomitant procedures in isolation. Consequently, the benefit SURD-AVR affords these patients remains unknown [20].

Whilst long-term data comparing SAVR or TAVR with Perceval Valve implantation has been lacking, Muneretto et al. has provided the first insights into its mid-term capabilities against TAVI [24]. After propensity matching, they demonstrated superior post-operative mortality and significantly less peripheral vascular complications and PPI in the Perceval Valve group [24]. At 5-year follow up significantly less mortality and major adverse cardiovascular and cerebrovascular events (MACCE), lower mean gradients and reduced PVL were seen in the Perceval Valve group [24]. Similar findings have been echoed in a recent meta-analysis of ten comparative studies published by Sa et al. [80].However, haemodynamic assessment was limited to post-operative outcomes, with only long-term PVL and mortality data pooled. Furthermore, only one of their included studies recorded 5-year mortality data, whilst two studies only provided data beyond two years, with four of the studies providing no data beyond twelve months [80]. Subsequently, comparative data evaluating mid-long term haemodynamic outcomes remains scarce. Supplementing this new wave of comparative data is the Perceval Sutureless Implant Versus Standard Aortic Valve Replacement Trial (PERSIST-AVR trial), comparing SAVR with Perceval Valve implantation, which has demonstrated promising 12-month results [81, 82]. This blinded multi-centre RCT has demonstrated non-inferior haemodynamics, mortality and (MACCE) at 12 months, with significantly reduced cross-clamp and bypass times [81, 82]. The results from PERSIST-AVR are promising and with encouraging mid-term data demonstrating acceptable mortality, morbidity and hemodynamic outcomes, surgeons may have increased confidence with the performance of this valve.

Promisingly, recent evidence has evaluated extended indications for SURD-AVR for both Perceval and Intuity Valves. Due to abnormal annular geometry posed by Bicuspid Aortic Valves (BAV), concerns regarding paravalvular leakage have resulted in its contra-indication in those with BAV. Several studies have demonstrated acceptable ACC times between 40 and 70 min, CPB times between 50 and 80 min and perioperative mortality between 0–2% with no studies identifying major PVL at discharge [83–85]. However, one study evaluating the Intuity Valve’s mid to long-term performance demonstrated a significantly higher incidence of PVL, with 10% of BAV patients having severe PVL compared with only 3% in the TAV group, although this did not have a significant influence on reoperation or mortality [86]. Other indications such as aortic regurgitation have been evaluated recently with acceptable operative and short-term outcome data demonstrated in a small cohort of patients, however long-term data is lacking [87]. Whilst promising, this data has to be interpreted with caution. All studies were retrospective and evaluated small cohorts of patients, and only one evaluated mid-term data. Further research will be required before the indications for SURD-AVR can be extended.

Limitations in this study require consideration when interpreting the findings. Five of the seven studies were non-randomised, single arm observational trials [16, 21, 25–27]. Due to their design, confounders were unable to be accounted for. These factors, in conjunction with their unclear recruitment strategies, insufficient reporting of key outcome data and reported financial conflicts of interest, introduce significant selection and reporting bias into the sample. However, at least with respect to short term data, this is lessened by the large sample sizes and wide recruitment base. Whilst the long-term data is promising, modest numbers at long-term follow-up as well as absence of loss-to follow-up reporting in three studies, adds attrition bias into this study. The significant heterogeneity encountered in key outcomes, whilst likely explained by differences in procedural approach, population size and variation in concomitant procedures, limits the confidence in some of the above findings. Additionally, despite patients undergoing concomitant procedures or those with small aortic roots identified as two key groups likely to benefit from reduced surgical times offered by implantation of this valve, no short or long-term data was available for either at the time of this review. This leaves a significant, unanswered gap in the literature for these groups. Long-term randomised control trials will be required to affirm the findings in this study.

## Conclusions

This is the first systematic review and meta-analysis evaluating the mid-term outcomes and durability of the Perceval S Valve in isolation. The analysis has demonstrated that the Perceval Valve has acceptable haemodynamics, durability and mortality with acceptable freedom from mortality at mid-term follow-up. However, future long-term, randomised, comparative data will be required to better characterise the Perceval Valve’s clinical outcomes.

### Supplementary Information

**Additional file 1.**

## Data Availability

All data generated or analysed during this study are included in this published article. If raw data is required, this can be made available upon request to the corresponding author.

## Appendix A: Example MeSH keywords and search strings

1. Sutureless Aortic Valve
2. Sutureless AND Aortic Valve Stenosis
3. Sutureless AND Aortic Valve Replacement OR AVR
4. Perceval Valve AND Aortic Valve Replacement OR AVR
5. Perceval Valve AND Aortic Valve Stenosis OR Aortic valve stenosis OR Aortic Valve OR Aortic valve
6. Aortic Stenosis or Aortic Valve Stenosis
7. SURD OR SURD-AVR OR SURD-Aortic Valve Replacement
8. Rapid-Deployment AND Aortic Valve Stenosis
