# Supplementary material for: Perceval valve intermediate outcomes: a systematic review and meta-analysis at 5-year follow-up
Source: J Cardiothorac Surg. 2023 Apr 11;18:129. doi: 10.1186/s13019-023-02273-7 (PMC10091543; doi:10.1186/s13019-023-02273-7)
Supplement: Supplementary file 1 — Additional file 1. [file 13019_2023_2273_MOESM1_ESM.docx]

|  | **Bias due to Confounding** | **Bias in Selection** | **Bias in Classification of Interventions** | **Bias due to deviation from intended interventions** | **Bias due to Missing Data** | **Bias in measurement of Outcomes** | **Bias in Selection of reported result** | **Overall** |
| --- | --- | --- | --- | --- | --- | --- | --- | --- |
| **Meuris et al 2016** | Severe | Moderate | Low | Moderate | Low | Moderate | Moderate | Severe |
| **FIschlein et al 2021** | Severe | Low | Low | Moderate | Low | Moderate | Moderate | Severe |
| **Glauber et al 2020** | Severe | Moderate | Low | Moderate | Severe | Moderate | Moderate | Severe |
| **Muneretto et al 2022** | Low | Low | Low | Moderate | Moderate | Moderate | Moderate | Moderate |
| **White et al 2022** | Low | High | Low | Moderate | Low | High | High | Severe |
| **Szecel et al 2021** | Severe | High | Low | Moderate | Low | Moderate | Moderate | Severe |
| **Lamberigts et al 2022** | Severe | High | Low | Moderate | High | Moderate | High | Severe |

**Supplemental Table 1: ROBINS-I Risk of Bias Assessment**
